# ChemBank Coming Soon and the Latest Great Flood Claims Research

**DOI:** 10.1100/tsw.2001.66

**Published:** 2001-06-22

**Authors:** Shauna Haley

## Abstract

Nature leads the news week with a report on a new database initiative designed to catalog the effects of small molecules on proteins. Tropical Storm Allison's widespread destruction of research at Texas universities tops the news in Science.

The U.S. National Cancer Institute (NCI) has announced plans to develop an open-access database tying chemicals to their effects on proteins, reports *Nature*. The data would be compiled from scientists willing to contribute findings about the molecular interactions between chemicals and proteins. Also to be included in the project is data about how molecules affect an organism's phenotype. To get its database jump-started, the NCI will open several labs this year that churn out molecules and test their biological activities. Several facilities are being considered to host the labs and will curry the heavyweight support of the NCI. *Nature* notes that the so-called "ChemBank", if successful, would house all existing data of the emerging field of chemical genetics. One scientist quoted by the journal observes that researchers now must datamine among disparate sources for such information in the literature, and that the database would streamline molecular discovery efforts.

Houston, Texas may seem an unlikely place to pool the largest sprawl of medical research complexes in the world, but University of Texas M. D. Anderson Cancer Center, Medical School at Houston and the Baylor College of Medicine together represent a unified powerhouse of scientific research. *Science* this week reports on the devastation of these facilities caused by Tropical Storm Allison in the second week of June this year. *Nature* also carries the story in third position. The storm soaked the campuses on June 7, says *Science*, but the real damage came when Allison returned unexpectedly a few days later to dump 36 centimeters more rain on Houston. Such catastrophic flooding occurs about once a century, notes the journal. Tens of thousands of specialized research animals were drowned, tissue and cultures in freezers destroyed, as well as many pieces of expensive equipment – such as several electron microscopes – were lost in the floodwaters and resulting power outages. Transgenic murine models that took 10 years or more to create were lost in the storm's fury and whole herds of animals will have to be bred. The cleanup of the carcasses has been left to the researchers, who, in biohazard suits, descend into their labs to haul away ruined experiments and animals – time lost the researchers' consequence of natural disaster.
